# Maimendong Decoction Improves Pulmonary Function in Rats With Idiopathic Pulmonary Fibrosis by Inhibiting Endoplasmic Reticulum Stress in AECIIs

**DOI:** 10.3389/fphar.2020.01262

**Published:** 2020-08-14

**Authors:** Mengmeng Shen, Yanan Nan, Lan Zhang, Liming Di, Shuangshuang He, Yu Li, Yadong Li

**Affiliations:** ^1^ School of Traditional Chinese Medicine, Beijing University of Chinese Medicine, Beijing, China; ^2^ School of Life Sciences, Beijing University of Chinese Medicine, Beijing, China

**Keywords:** Maimendong decoction, pulmonary function, pulmonary fibrosis, endoplasmic reticulum stress, alveolar type II epithelial cells

## Abstract

This study was designed to investigate the mechanism by which MMDD improves lung function, and observe the effect of MMDD on endoplasmic reticulum stress(ERS) in alveolar type II epithelial cells (AECIIs) of pulmonary fibrosis rats. pulmonary fibrosis animal model was established by intratracheal injection of BLM at a dose of 6mg/kg body weight. Overall, Thirty male SPF Sprague-Dawley rats were randomly divided into control group, BLM group and BLM+MMDD group. BLM+MMDD group rats were fed 24 g/kg over three weeks for twice a day on the fourteenth day after model establishment. MMDD improves pulmonary function of fibrotic rats and reduces the occurrence of endoplasmic reticulum stress in AECIIs. MMDD could significantly improve the forced vital capacity (FVC) of bleomycin-induced pulmonary fibrosis in rats. MMDD reduced the expression of GRP78 and CHOP in AECIIs, increased the secretion of surfactant protein C (SPC) by AECIIs. Moreover, the apoptosis of the fibrosis zone in the lung tissue was remarkably mitigated by administration of MMDD. The finding of this study revealed that MMDD can improve lung function in rats with pulmonary fibrosis by reducing the occurrence of ERS and cell apoptosis of AECIIs. It may provide a new method for the treatment of pulmonary fibrosis.

## Introduction

IPF is a chronic progressive fibrotic interstitial pneumonia. The lesions caused by IPF are localized in the lungs and occur in middle-aged and elderly men. The symptoms of IPF are mainly manifested by progressively worsened breathing difficulties, with restricted ventilation dysfunction and gas exchange. IPF has a poor prognosis, as it, leads to hypoxemia and respiratory failure. The lung histology and chest high-resolution CT (HRCT) of IPF appear as common interstitial pneumonia (UIP) (Raghu et al., 2015). IPF is a terminal lung disease with pathological manifestations of alveolar structural and its epithelial damage or collapse, and activation and proliferation of lung fibroblasts, and excessive accumulation of extracellular matrix, causing an increased pulmonary interstitial and decreased lung function (Longo et al., 2018). The pathogenesis of pulmonary fibrosis is currently unclear. In recent years, many studies have shown that endoplasmic reticulum stress (ERS) plays an important role in the development of IPF (Korfei et al., 2008; Hsu et al., 2017). These studies have demonstrated that IPF is more sensitive to ERS. ERS can damage AECIIs, and it is more prone to abnormal AECIIs epithelial repairing and processing, which accelerates the development of pulmonary fibrosis (Korfei et al., 2008).

In recent years, with the understanding of pathogenesis of pulmonary fibrosis in Chinese medicine, some progress has been made in the treatment of pulmonary fibrosis with traditional Chinese medicine (Ying et al., 2016; Yunping et al., 2018; Xiaolin et al., 2018). MMDD which consists of the roots consisted of six herbs (Ophiopogonis Radix, Pinelliae Rhizoma Praeparatum Cum Alumine, Ginseng Radix et Rhizoma, Glycyrrhizae Radix et Rhizoma Praeparata Cum Melle, Oryza sativa subsp. japonica S.Kato, Jujubae Fructus) has a variety of pharmacological effects such as regulating the secretion of multi-alveolar surfactant, antitussive, promoting airway purification, and relieving the high sensitivity of the respiratory tract (Wang and Ji, 2016). In addition, MMDD also treats radiation pneumonitis, allergic asthma, chronic bronchitis with lung yin deficiency, and pulmonary fibrosis (Lin et al., 2007; Luo, 2013; Yu, 2015; Kang et al., 2017). Considering the properties of those herbs, and based on the previous research results of our team, this study used MMDD to treat pulmonary fibrosis and observe its effect on ERS. Several active components of MMDD have been shown to be bioactive *in vivo*. Ophiopogonis Radix (Ophiopogon root), which nourishes the yin, has been used in clinical practice to promote fluid secretion and to moisturize the lungs and skin in traditional Chinese Medicine. There are many extractions of *Ophiopogon Radix* also have the anti-inflammatory and anti-apoptotic effects, such as Steroidal saponins, 4′-O-Demethylophiopogonanone E and methylophiopogonanone A (He et al., 2016; Zhao et al., 2017; Wu et al., 2019). Total ginsenosides extract could enhance autophagy flux and induce autophagic cell death through activation of ERS. Emerging evidence demonstrated this process was mediated by the ATF4-CHOP-AKT1-mTOR axis in NSCLC cells (He et al., 2016; Zhao et al., 2019); and Liquiritin had the effects of anti-inflammation, anti-oxidative stress, and anti-cell apoptosis in a rat model (Luo et al., 2019).

In this study, we examined the pulmonary function, pulmonary histomorphology, collagen content, ERS, and cell apoptosis in AECIIs of pulmonary fibrosis rats after administration of MMDD. The purpose of this study was aimed to observe the mechanism by which MMDD improves lung function, and relieves the ERS in AECIIs.

## Materials and Methods

### Reagents

Bleomycin (BLM) was purchased from Nippon Kayaku (Batch number 970592). GRP78/Bip rabbit polyclonal antibody (catalog number:11587-1-AP), GAPDH mouse monoclonal antibody (catalog number: 60004-1-Ig), CHOP/GADD153 mouse monoclonal antibody (catalog number:60304-1-Ig), SFTPC polyclonal antibody (catalog number:10774-1-AP), and CHOP rabbit polyclonal antibody (catalog number:115204-1-AP) were purchased from Proteintech, America.

### Sample Preparation and Constituents Identification of MMDD

Ophiopogonis Radix, Pinelliae Rhizoma Praeparatum Cum Alumine, Ginseng Radix et Rhizoma, Glycyrrhizae Radix et Rhizoma Praeparata Cum Melle, Jujubae Fructus were produced by Beijing Tongrentang (Bozhou) Yinpian Co., Ltd. (Anhui, China) and purchased from Guoyi Tang of Beijing University of Chinese Medicine (Beijing, China). Oryza sativa subsp. japonica S.Kato was purchased from Kaiyuan Jefu Trading Company. samples were identified by professor Chunsheng Liu (Voucher numbers: 700003252; 601232835; 501002767; 171103007; 700003080; SC10121128200214). Detailed information of the drug materials and the scan of the vouchers were given in **Supplementary Table 1**. The voucher specimens were deposited in School of Chinese Materia Medica, Beijing University of Chinese Medicine. MMDD derived from *Jinkuiyaolue*, a classic medical book. According to the traditional decoction method of modern Chinese medicine hospital, 42 g of Ophiopogonis Radix, 6 g of Pinelliae Rhizoma Praeparatum Cum Alumine, 1.5 g of Glycyrrhizae Radix et Rhizoma Praeparata Cum Melle, 1.5 g of Ginseng Radix et Rhizoma, 2.5 g of Oryza sativa subsp. japonica S.Kato, 1.8 g of Jujubae Fructus were weighed respectively. all the drugs were mixed together, Soaking in 700 ml deionized water for 30 min, then all the soaked medicine and water were poured into the electric casserole, selected the big fire for 45 min, simmer for 20 min the MMDD decoction were concentrated to 2 g/ml for further use.

### LC/MS Conditions

In order to ensure the quality of the drug, the main components of the six herbs of MMDD were analyzed by Ultra Performance Lquid Cromatography/Mss Sctrometry (UPLC/MS). MMDD decoction (100 μl) was taken from the filtrate of the broth, and 1 ml (methanol: water = 1:1) was added, and the mixture was vortexed for 1 h, and centrifuged, and the supernatant was taken for injection. UPLC analysis was performed with a Waters (ThermoFisher, MA, USA) Rapid Separation LC system equipped with a waters UPLC HSS T3 (1.8 um 2.1 mm*100 mm). A gradient elution program was conducted for chromatographic separation with mobile phase A (water 0.1% formic acid) and mobile phase B (acetonitrile) as **Supplementary Table 2**. The flow rate was 0.3 ml/min, the injection volume was 1.0 μl, and the column temperature was 50°C.

The mass spectrometry was performed using a quadrupole orbital ion trap mass spectrometer equipped with a thermoelectric spray ion source. The positive ion source voltages were 3.7 kV, respectively. The capillary heating temperature was 320°C. The plenum pressure is 30 psi and the auxiliary gas pressure is 10 psi. The volume is heated and evaporated to a temperature of 300°C. Both the updraft and the auxiliary gas are nitrogen. The collision gas was nitrogen and the pressure was 1.5 m Torr. The first-level full scan parameters are: resolution 70000, automatic gain control target of 1 × 10^6^, maximum isolation time of 50ms, and mass-to-charge ratio scanning range of 50–1,500. The liquid quality system is controlled by Xcalibur 2.2 SP1.48 software, and data acquisition and targeted metabolite quantification are performed by the software.

### Animals

Male Sprague-Dawley (SD) rats (380 ± 20g) were obtained from Beijing Vital River Laboratory Animal Technology Co., Ltd.(Beijing, China, animal certificate number: SCXK(Beijing) 2016-0006). The animal studies were approved by the Animal Ethics Committee of Beijing University of Traditional Chinese Medicine(BUCM-4-2017101301-402). The laboratory temperature was maintained at 22.9 degrees Celsius (°C), and relative humidity at 46.4%, and 12 h dark-light cycle. All rats were fed with standard food and water ad libitum in the laboratory for 14 days before experiment.

### Model Preparation and Administrations

After 14 days of adoption, all rats were randomly divided into control group (n=10), BLM group (n=10), BLM+MMDD group (n=10). To establish the pulmonary fibrosis model, the control group treated with nothing, the other groups rats were intratracheally injected with bleomycin (6 mg/kg) dissolved in saline. The control group and BLM group rats were tread by saline, and the BLM+MMDD group rats were given MMDD was administered at a dose of 2.4 g/100 g body weight via oral gavage administration for 21 days. All the treatments were initiated 14 days after bleomycin established a pulmonary fibrosis model, twice a day for 21 days. All the rats were sacrificed on day 35.

The rats were anesthetized with 1% sodium pentobarbital, lung function test was performed first, and then left and right lung tissues of the rats were taken. The rats were anesthetized with 1% sodium pentobarbital, lung function tests were performed first, and then left and right lung tissues of the rats were taken. The left lung is centered on the hilum and divided into three parts. The lower 1/3 of the left lung, which was fixed with 10% neutral formalin solution, was treated with HE staining, MASSON staining, immunohistochemical staining, immunofluorescent staining, and TUNEL staining. The middle 1/3 and upper 1/3 of the left lung were stored at -80°C, which were used for WB analysis and HYP content determination, respectively.

### Pulmonary Function Test

1% sodium pentobarbital (70 mg/kg) was anesthetized by intraperitoneal injection. The tracheal intubation was performed after tracheotomy, and the intubation was fixed with the trachea. The rat was placed supine in a medium-sized body drawing box, connected to a breathing machine, and set a breathing ratio of 20:10 and a respiratory rate of 65 times/min. The forced vital capacity (FVC) and dynamic pulmonary compliance (Cdyn) were measured by a 30 cm H_2_O pressure. FVC was tested by ventilator (Bestlab, Beijing).

### Hydroxyproline Assay in Lung Tissue

HYP contents were measured according to the manufacture’s instruction of the kit (Jiancheng, Nanjing, China). Lung tissues (100 mg) were hydrolyzed with 750 ul akaline hydrolysate and high pressure at 120°C for 60 min and then centrifuged at 5,000 rpm for 10 min at 4°C. The supernatant was obtained, and hydroxyproline content was measured on an ultraviolet spectrophotometer (Thermo, US). Results were expressed in microgram per gram tissue (ug/g tissue).

### Histomorphology and Immunohistochemical Analysis

The lower 1/3 of the left lung was fixed with 10% neutral formalin solution, paraffin-embedded, coronal section, standard hematoxylin and eosin (HE) solution (Solarbio, Beijing, China) and Masson’s trichrome staining kit (Solarbio, Beijing, China) and immunohistochemical staining (Boster, Beijing, China) was carried out to observe and photograph. Immunohistochemical staining was performed strictly according to the instructions of the ready-to-use immunohistochemistry kit. Conventional baking, dewaxing, antigen inactivation, heat repair of antigen, and blocking endogenous peroxides. Primary antibody: rabbit-derived GRP78(1:1,000), CHOP antibody (1:200) and SPC antibody (1:300) were incubated overnight at 4°C; After washing the slides thrice with PBS, the sections were then incubated with secondary antibody for 20 min at 35°C. Sections were then washed with PBS and incubated with DAB for 10 min. Nuclei were counterstained with hematoxylin. Positive staining for GRP78, CHOP and SPC were brown. Expression of GRP78, CHOP, and SPC was compared between groups by calculating the ratio of positive staining area.

### Immunofluorescence Staining

The paraffin sections were baked in a constant temperature oven at 60°C for 2 h, gradient dewaxing with xylene and alcohol. Endogenous performed activity was blocked in a solution of 3% hydrogen peroxide. Antigen retrieval by microwave heating with 0.1 M sodium citrate buffer (pH 6.0), 0.3% Titron X solution diluted with 0.1 M PBS was added to the sections and placed in a wet box, and allowed to stand at room temperature for 30 min; 5% normal goat serum was added dropwise, and the mixture was sealed at room temperature for 30 min to remove excess liquid, and the primary antibodies of GRP78 (1:100) and SPC (1:100) were incubated overnight at 4°C. After washing the slides three times with PBS, the sections were then incubated with secondary antibody for 20 min at room temperature, the fluorescently labeled secondary antibody (1:100) was added dropwise and incubated for 1 h at room temperature, then nuclei were counterstained with DAPI (1 ug/ml). The sections were sealed with anti-fluorescence quencher.

### Western Blotting Analysis

100 mg left lung upper lobe tissue was placed in a glass grinder, 200 μl RIPA lysate and 2 μl PMSF were added per 20 mg, and the tissue homogenate was repeatedly ground on ice. After centrifugation (12,000 r/min, 10 min at 4°C), the supernatant was collected, packed, and stored in -80°C. Just before using, the loading buffer was mixed into the supernatant at a ratio of 1:4 and cooked in boiling water for 10 min. Proteins in the supernatant were separated by SDS-PAGE on a 10% SDS polyacrylamide gel, 20 μg protein sample per well was loaded, and then transferred to polyvinylidene fluoride (PVDF) membranes. The blotted membranes were blocked with 5% non-fat dairy milk (w/v). Blotted membranes were washed by 0.1% Tween-TBS (TBST) three times (10 min per time) after 2 h. Then blotted membranes were incubated at 4°C overnight with primary anti-GRP78 antibodies (1:1,500, Proteintech, Chicago, USA), anti-chop antibodies (1:300, Proteintech, Chicago, USA) and with secondary antibodies at room temperature for 1 h. After being washed with TBST three times (10 min per time), the blots were visualized with ECL reagent (MilliPore, Massachusetts, USA). Protein levels were analyzed using Image Lab software. Gray-scale analysis of the target strip was performed using Image J software to calculate the relative integrated optical density value.

### TUNEL Staining

Apoptotic cells were visualized by terminal deoxynucleo-tidyl transferase-mediated dUTP nick end labeling (TUNEL) staining according to the manufacturer’s instructions (Key Gen biological Technology, Nanjing, China). The paraffin sections were baked in a constant temperature oven at 60°C for 2 h, gradient dewaxing with xylene and alcohol; the sections were digested with proteinase K and washed three times with 1× PBS. After that, the sections were incubated with 50 μl terminal deoxynucleotidyl transferase (TdT) enzyme at 37°C for 60 min in a wet box. At last, streptavidin-labeled horseradish peroxidase (HPR) were added in a wet box at 37°C for 60 min, then the nuclei were counterstained with DAPI (1 ug/ml). Sealing was with anti-fluorescence quencher.

### Statistical Analysis

All the data were statistically analyzed using SPSS20.0 software (IBM, NY, USA), and the normal measurement data conforming to the normal distribution was expressed by the mean ± SEM, Kruskal-Wallis *H* test was used if the data do not comply with the normal distribution; if the data comply with the normal distribution, one-way analysis of variance followed by SNK or Dunnett’s T3 *post hoc* test was used for multiple comparisons. Differences were considered to be significant at *P* values < 0.05.

## Results

### The Result of LC/MS

**Figure 1:**
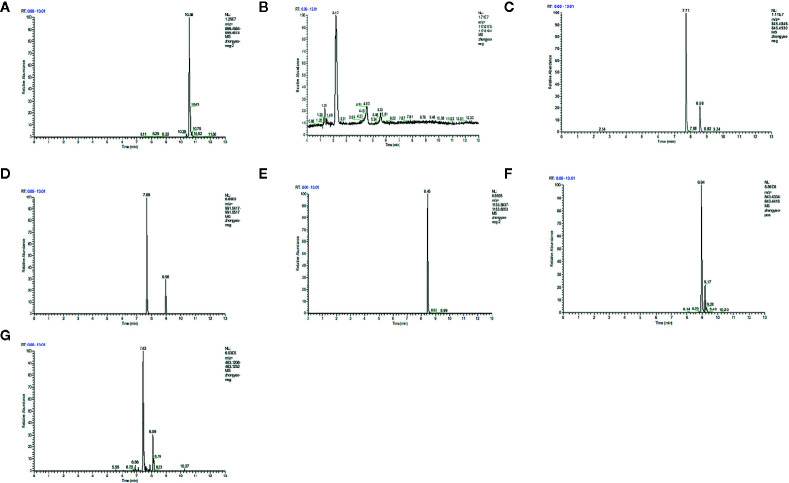
LC/MS chromatogram map. **(A)** ophiopogonin; **(B)** Succinate; **(C)** ginsenoside Rg1; **(D)** ginsenoside Re; **(E)** Ginsenoside Rb1; **(F)** Ammonium glycyrrhizinate; **(G)** Liquiritin.

LC/MS was used to analyze the main components of MMDD. Five major components, including ophiopogonin D, Succinate, ginsenoside Rg1, ginsenoside Re, Ginsenoside Rb1, Ammonium glycyrrhizinate, Liquiritin were identified in MMDD (**Figure 1**).

### MMDD Ameliorated Pulmonary Function in Rats

**Figure 2:**
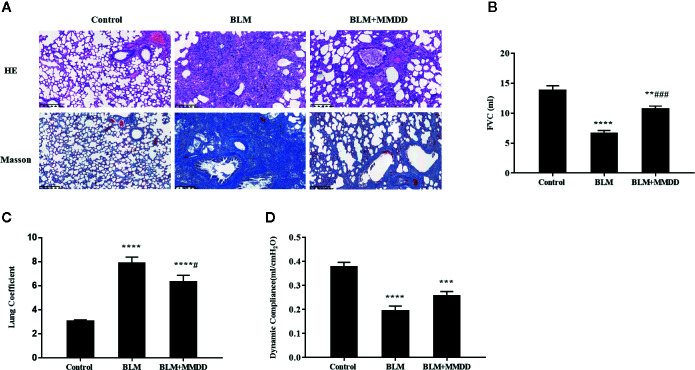
Effect of MMDD on histopathologic changes and Pulmonary Function in BLM-induced pulmonary fibrosis rats. **(A)** All sections were stained with HE and representative sections are shown at the same magnification. All sections were stained with HE and representative sections were shown at the same magnification. Bars: 50 μm. **(B)** FVC; **(C)** Lung coefficient; **(D)** Dynamic compliance. Data were analyzed by one way ANOVA (Mean ± SEM). **P < 0.01, ***P < 0.001, ****P < 0.0001, compared with the control group. ^#^P < 0.05, ^###^P < 0.001, compared with the BLM group.

As we know, progressive aggravation of pulmonary function is a feature of IPF. To elucidate the effects of MMDD on BLM-induced pulmonary dysfunction, pulmonary function test was tested dynamically. We observed that compared with control group, FVC, FVC/weight and Cdyn of the BLM group were significantly decreased (**Figures 2B–D**). After using MMDD, FVC, lung coefficient, and lung compliance were increased significantly in comparison with the BLM group rats. These data indicated that MMDD improved the lung function induced by BLM in fibrotic rats.

### MMDD Alleviated Pulmonary Injuries in Rats

o identify the degree of lung injury after treatment, sections of lung tissue were stained with H&E and Masson trichrome. The infiltration of numerous inflammatory cells was observed in the alveolar and interstitial space of the lung by HE staining, with the lung tissue of the BLM group was extensively transformed, the alveolar structure was disordered or collapsed, and the alveolar septum was relatively thicker (**Figure 2A**). In the BLM+MMDD group, apparent alleviation of pulmonary alveolitis was observed. Pulmonary fibrosis zone was reduced largely, the alveolar structure was gradually healed, the alveolar septum was thinner, and the number of interstitial inflammatory cells was significantly reduced. Additionally, the Masson staining showed that blue-stained collagen was massively increased in the BLM group. The collagen deposit of BLM+MMDD group was alleviated significantly according to the result of the Masson staining.

### MMDD Reduced Collagen Content in Lung Tissue of Fibrotic Rats

**Figure 3:**
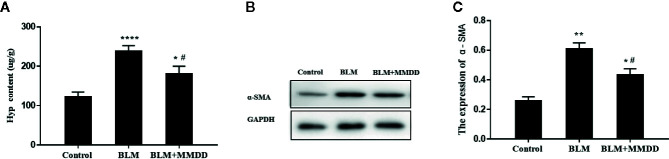
MMDD reduced collagen content in lung tissue of Fibrotic rats. Data were analyzed by one way ANOVA (Mean ± SEM). **(A)** HYP content in lung tissue of rats in each group; **(B)** The expression level of the α-SMA was detected by Western blot analysis; **(C)** The quantitative result of the α-SMA expression. *P < 0.05,**P < 0.01, ****P < 0.0001, compared with the control group. ^#^P < 0.05, compared with the BLM group.

Pulmonary fibrosis was characterized by collagen accumulation. HYP was an index of collagen contents. Analyses of HYP contents were conducted to evaluate the effect of MMDD formula (**Figure 3A**). Compared with the BLM group, The contents of HYP were significantly decreased. in the BLM+MMDD group (*P*<0.05). The expression of α-SMA was tested by Western blot analysis (**Figure 3B**). After modeling, the level of α-SMA was significantly increased compared with control group. In the BLM+MMDD group, the expression of the level of α-SMA was much lower than the BLM groups (**Figure 3C**).

### Results of Immunohistochemistry Staining for GRP78, CHOP, and SPC

**Figure 4:**
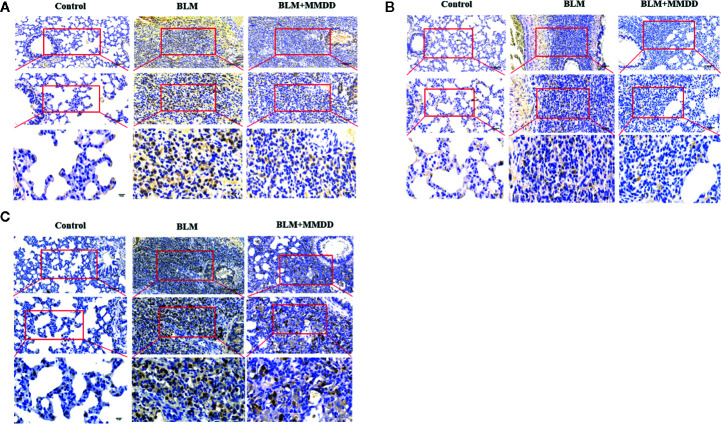
Effect of MMDD on the expression of GRP78, CHOP, and surface active protein C (SPC) in the lung tissue of BLM-induced pulmonary fibrosis rat by immunohistochemical analysis. **(A)** The expression of GRP78 in each groups; **(B)** The expression of CHOP in each groups; **(C)** The expression of SPC in each groups. All the sections were detected by immunohistochemical analysis. Bars: (upper panels: 100 μm; middle panels: 50 μm; lower panels: 10 μm.)

In **Figures 4A, B**, compared with the control group, the expression of GRP78 and CHOP was markedly increased in the BLM group, and Positive cells are stained brownish yellow. When compared with BLM group, the expression of GRP78 and CHOP could be inhibited in groups that were treated with MMDD by immunohistochemistry testing. The SPC synthesized and secreted by AECIIs in rat normal lung tissue was evenly distributed on the alveolar surface. In lung tissue, only AECIIs secreted SP-C. As shown in **Figure 4C**, the SPC immunohistochemical staining was light brown. Compared with the control group, the SPC was observed in the fibrosis zone of the lung tissue of the BLM group. The brown color was strongly positively expressed, and its distribution was mainly confined to AECIIs. At the same time, the cytoplasm of AECIIs was significantly vacuolated. Compared with the BLM group, SPC retained in AECIIs of BLM + MMDD group was decreased, and SPC secreted to the alveolar surface was increased. The Positive expression of GRP78 and CHOP indicated that ERS occurred in pulmonary fibrosis caused by bleomycin. AECIIs secreted more SPC to the surface of the alveoli, which contributed to the alveolar contraction and stability.

### Expression of GRP78, CHOP, and SPC in Lung Tissues

**Figure 5:**
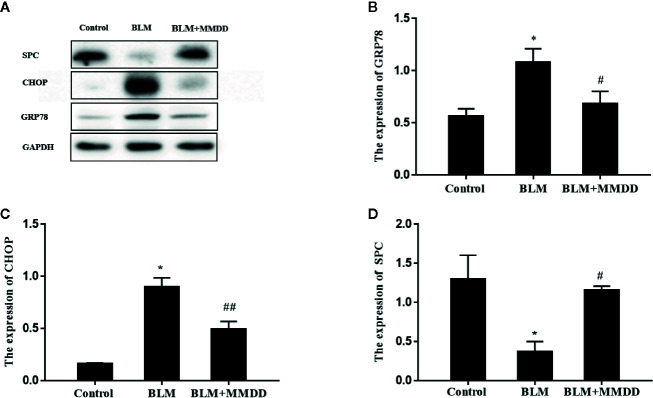
Effect of MMDD on the expression of GRP78 and CHOP in the lung tissues of BLM-induced pulmonary fibrosis rat by Western blot analysis. **(A)** The expression of GRP78, CHOP and surface active protein C (SPC) in the lung tissues were detected by Western blot analysis; **(B)** The quantitative result of the GRP78 expression; **(C)** The quantitative result of the CHOP expression; **(D)** The quantitative result of the SPC expression. Data analyzed by one way ANOVA (Mean ± SEM). *P < 0.05, compared with the control group. ^#^P < 0.05, ^##^P < 0.01,compared with the BLM group.

The expression levels of GRP78, CHOP and SPC were tested by Western blot analysis (**Figure 5**). After modeling, when compared to the control group, the expression levels of GRP78 and CHOP were obviously increased, while the expression of SPC was significantly decreased. Compared with the BLM group, the expression levels of GRP78 and CHOP of the BLM+MMDD group were significantly decreased (*P*<0.05). In contrast, the expression of SPC was increased. These findings revealed that ERS was increased and the secretion of SPC was significantly reduced in the model of bleomycin-induced pulmonary fibrosis. After treatment with MMDD, endoplasmic reticulum stress was relieved, and the secretion of SPC in AECIIs was enhanced.

### Results of Immunohistochemistry Staining for GRP78, CHOP, and SPC

**Figure 6:**
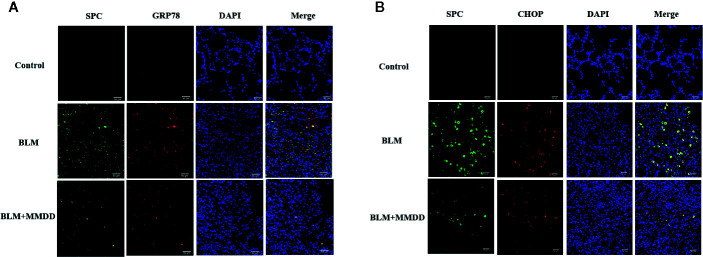
Effect of MMDD on the expression of GRP78, CHOP, and surface active protein C (SPC) in the lung tissue of BLM-induced pulmonary fibrosis rat by Immunofluorescence double staining. **(A)** Immunofluorescence staining images for detection of CY3 (red), FITC (green) expression. **(B)** Immunofluorescence staining images for detection of CY3 (red), FITC (green) expression. DAPI (blue) was used for staining of nuclei. Bars: 50μm.

From the results of immunohistochemistry, we found that GRP78, CHOP and SPC were expressed in the cytoplasm of the cells, so we used co-localization detection of GRP78/SPC and CHOP/SPC by immunofluorescence double staining. we observed that GRP78 and SPC were expressed in the same cell. Since SPC is expressed only by AECIIs in lung tissue (**Figure 6A**), it can be confirmed that GRP78 and SPC are co-expressed in AECIIs. In addition, we also found that GRP78 was not only expressed in the cytoplasm of AECIIs, but also expressed in the cytoplasm of other cells, which cell is subject to further study. Similarly, we found that in the double staining of CHOP and SPC (**Figure 6B**), CHOP and SPC fluid were co-expressed in the AECIIs.

### Apoptosis of TUNEL Staining

**Figure 7:**
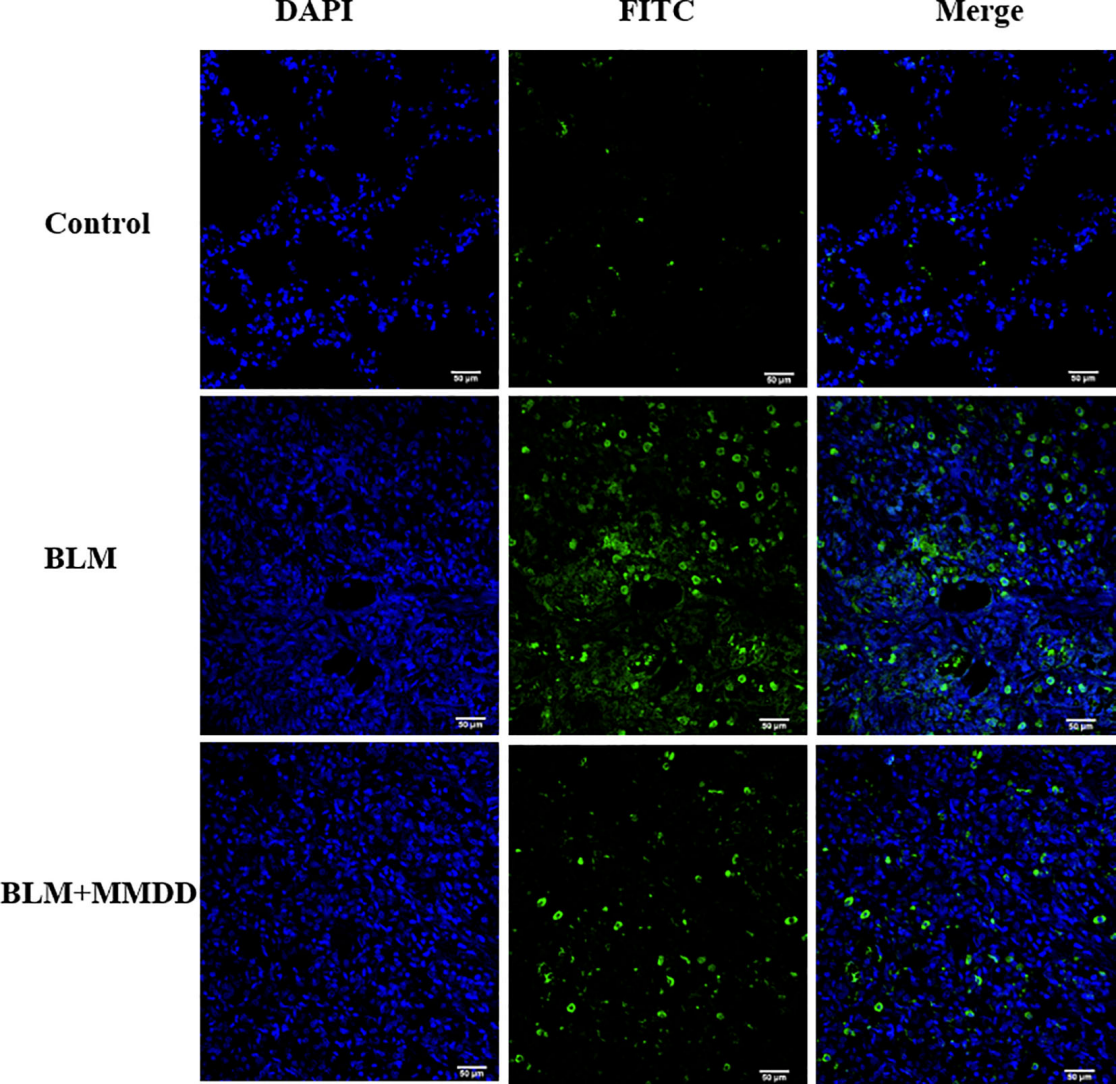
TUNEL staining of apoptotic cells in each group. Note: blue is the nucleus and green is the apoptotic cell. Bars: 50 μm.

There was almost no apoptosis in the lung tissue of the control group. Compared with the control group, the apoptotic cells in the fibrosis zone in the lung tissue of the model group increased. Compared with the BLM group, the number of apoptotic cells in the AECIIs of the BLM+MMDD group was significantly decreased (**Figure 7**).

## Discussion

In animal experimental research, BLM-induced pulmonary fibrosis rat or mouse models are generally used to study the pathogenesis and corresponding treatment methods (Moore and Hogaboam, 2008). Bleomycin has been used as an anti-tumor drug, but it is now thought to cause dose-dependent interstitial pulmonary fibrosis (Chen and Stubbe, 2005). Chen J has reported that BLM can activate endoplasmic reticulum stress-related proteins including GRP78, CHOP and ATF-4 *in vivo* and *in vitro*. When ERS inhibitors or Pakt inhibitors are used, lung fibroblasts are reduced and can effectively improve lung function (Zhang et al., 2017).

In the present study, our finding showed that MMDD not only delayed the pathological process of BLM-induced fibrotic rats, but also ameliorated the lung function of fibrotic rats. Those might be through reducing the inflammatory infiltration of the lung tissue of fibrotic rats, reducing the content of extracellular matrix collagen, and increasing the amount of active SPC secreted by AECIIs to the surface of the alveoli. Our previous studies have also shown that MMDD attenuated BLM-induced rat IPF by improving lung function and alleviating chronic inflammatory responses in fibrotic lung tissue (Kang et al., 2017).

Extensive extracellular matrix deposition in the lung septum is an important pathological feature of pulmonary fibrosis. Collagen is the main component of the extracellular matrix. HYP is a unique amino acid that makes up the body’s collagen. Except that elastin contains a small amount of HYP (about 1%), almost all HYP is present as collagen. Therefore, the HYP content in the tissue can directly reflect the degree of organ fibrosis. The direct source of collagen in the lung septum is secreted by activated myofibroblasts, while α-SMA is a special marker protein for myofibroblasts (Waisberg et al., 2012). Therefore, α-SMA is an indirect indicator of collagen content. So we used HYP and α-SMA as indicator to measure the degree of fibrosis in our research. Our experimental results showed that MMDD remarkably reduced the content of HYP and the expression of α-SMA in lung tissue of rats with BLM-induced pulmonary fibrosis. This might be one reason for the improvement of pulmonary function in fibrotic rats.

The alveoli are the terminal part of the lung bronchial bundle and the place where gas exchange takes place. It is the main structure that constitutes the lung. Alveolar epithelium is classified into AECIs and AECIIs according to their morphological structure and function (Dobbs et al., 1998). AECIIs are sites for the synthesis, storage and secretion of pulmonary surfactants (Fehrenbach, 2001; Schmitz and Müller, 1991). Alveolar surfactant is a secreted lipoprotein composed mainly of four related proteins, SP-A, SP-B, SP-C, and SP-D. Only AECIIs secrete SP-C. They have the function of reducing the surface tension of the alveoli, increasing the compliance of the lungs, maintaining the stability of the alveolar size, preventing atelectasis and preventing pulmonary edema. In addition, AECIIs also have the beneficial functions in alveolar fluid balance, cellular (epithelial) repair, removal of dead or apoptotic cells, immune regulation and host defense. William E et al. developed a transgenic mouse model by using the Tet-On system, in which the expression of the mutant L188Q SFTPC was induced. AECIIs are more susceptible to apoptosis after BLM treatment, and the presence of ERS in AECIIs enhances bleomycin-induced pulmonary fibrosis in mice (Lawson et al., 2011). In patients with sporadic IPF, Martina Korfei et al. observed that in patients with IPPD, severe ERS occurred in AECIIs in lung tissue of patients with IPF, and ERS participates in the pathogenesis of pulmonary fibrosis by inducing apoptosis of alveolar epithelial cells in patients with IPF (Korfei et al., 2008). In our study, our results show that the active SPC secreted by AECIIs was reduced in the rat model of pulmonary fibrosis, and most of the SPC was accumulated and existed in AECIIs in an inactive form. After treatment with MMDD, the active SPC secretion was increased. This may be another reason for the improvement of pulmonary function in fibrotic rats.

**Figure 8:**
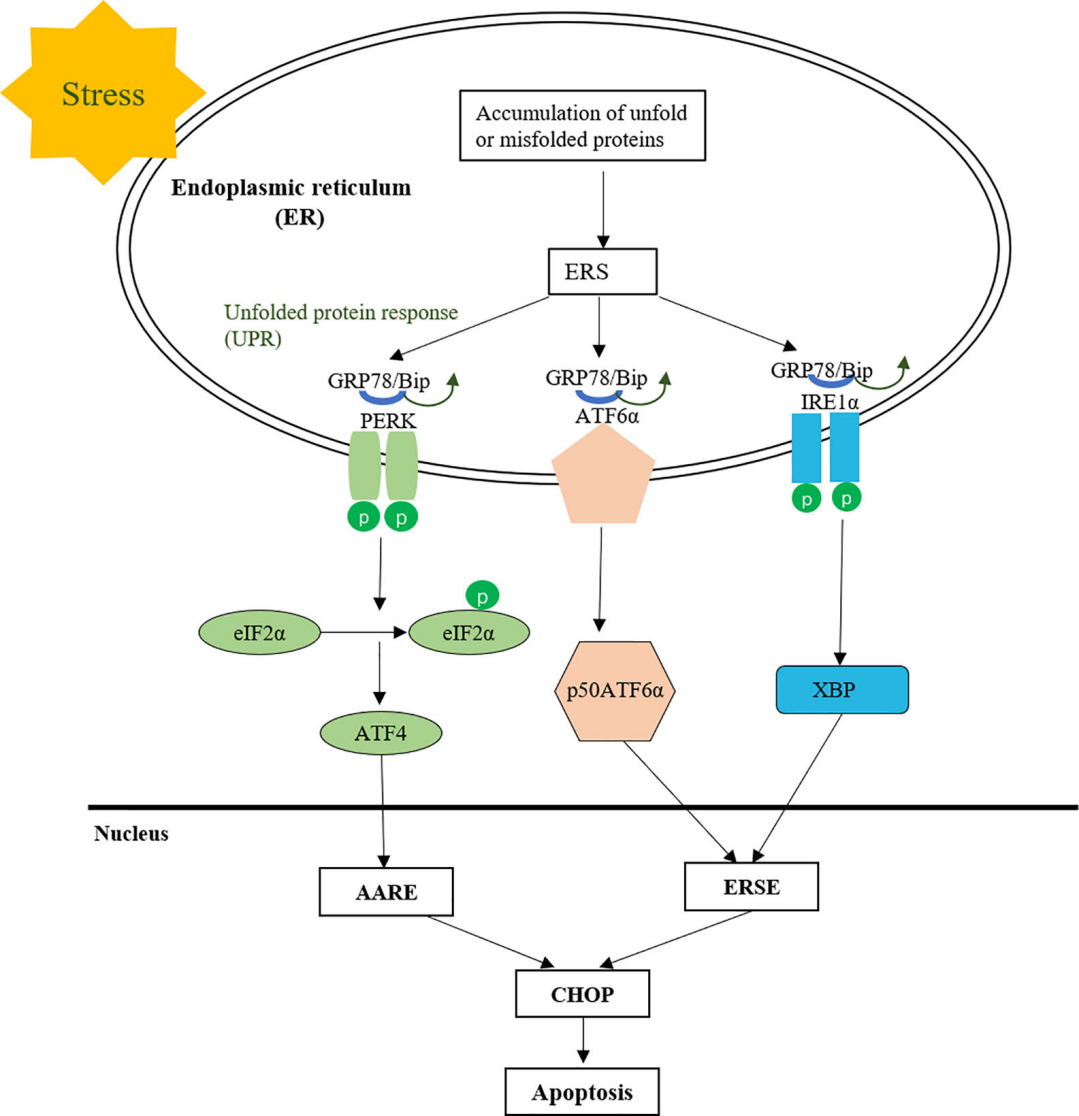
The protein processing in endoplasmic reticulum.

Furthermore, MMDD can reduce the occurrence of endoplasmic reticulum stress and apoptosis in AECIIs. Endoplasmic reticulum (ER) is an organelle responsible for the correct folding of membranes and secreted proteins, lipid biosynthesis, glycogen production and storage, and intracellular calcium homeostasis (Sevier and Kaiser, 2002; Tu, 2004). Under physiological conditions, the normal function of nascent proteins needs to undergo correct folding based on chaperone proteins and post-translational modifications are mainly aided by chaperone proteins such as the Hsp70 family members GRP78, calnexin and calreticulin (Ron and Walter, 2007; Saibil, 2013). However, under the action of various conditions that impair ER function, the accumulation of unfolded or misfolded proteins in the ER leads to the unfolded protein response (UPR) (Brown, 1996; Schröder et al., 2005). GRP78 is a molecular chaperone widely distributed in the endoplasmic reticulum, which is the main UPR regulator and promotes protein folding and prevents protein aggregation in ER, stabilizes protein conformation and improves ER folding, and serves as a quality control system for identifying, retaining, and ultimately eliminating targets (Welch and Brown, 1996). Normally, proteins involved in transmembrane are inactive with GRP78 in combination with protein kinase RNA-like endoplasmic reticulum kinase (PERK), activated transcription factor 6 (ATF-6), and inositol 1 (IRE-1). When UPR is activated, PERK, ATF-6, and IRE-1 are separated from GRP78, isolated GRP78 is involved in the correct folding of unfolded or misfolded proteins to reduce their accumulation in the endoplasmic reticulum cavity and reduce endoplasmic reticulum stress. If UPR does not rescue cells, the cells eventually undergo apoptosis through increased expression of CHOP or activation of ER-specific caspase, thus GRP78 or induction of CHOP is widely used as a marker for ER stress (**Figure 8**).

GRP78 immunoreactivity was observed in mouse AT2 cells after bleomycin injury, while overexpression of SPC - BRICHOS mutant SP-CDExon4 in A549 cells increased GRP78 (Zhong et al., 2011). After bleomycin was modeled, the expression of GRP78 was increased in rat lung tissue. These observations suggest that endoplasmic reticulum stress does occur in a bleomycin-induced rat fibrosis model. After treatment with MMDD, ERS in fibrotic rats was suppressed. CHOP is thought to represent a key regulator of pro-apoptotic responses under ER stress. Chop deficiency protects mice from BLM-induced lung injury and fibrosis (Yao et al., 2016). Our study found that the content of CHOP protein in lung tissue was significantly reduced after treatment with MMDD, indicating that MMDD can inhibit the development of pulmonary fibrosis through apoptosis of related cells. To determine whether ERS and apoptosis occurred in AECIIs, we used confocal immunofluorescent microscopy. Double-staining for GRP78 and SPC, CHOP, and SPC confirmed the increased ERS and apoptosis in AECIIs. In addition, TUNEL staining showed that MMDD can reduce the apoptosis of lung tissue cells in rats with pulmonary fibrosis lesions. Moreover, MMDD effectively inhibited the occurrence of ERS in AECIIs and the apoptosis of AECIIs.

There are three apoptotic pathways due to CHOP-mediated apoptosis. Therefore, the inhibition of apoptotic pathway and signaling pathway in fibrotic lung tissue by MMDD remain to be further studied. This study provides a basis for the study of traditional Chinese medicine compound, presents a new method for the treatment of pulmonary fibrosis, and established an experimental basis for exploring new targets for the treatment of pulmonary fibrosis.

## Conclusions

MMDD can mitigate the pathological development of pulmonary fibrosis. MMDD improved lung function by inhibiting the occurrence of ERS in AECIIs, reducing the apoptosis of AECIIs, and increasing the secretion of active SPC in AECIIs in lung tissue of fibrotic lesions. It may become a novel method for treating IPF.

## Data Availability Statement

The raw data supporting the conclusions of this article will be made available by the authors, without undue reservation, to any qualified researcher.

## Ethics Statement

The animal study was reviewed and approved by the Animal Ethics Committee of Beijing University of Traditional Chinese Medicine.

## Author Contributions

YaL and YuL designed this research. MS performed the animal experiments. LT and YN conducted the pulmonary function test. LZ, LD, and SH conducted LC/MS. MS conducted the biochemical experiments and analyzed the data. MS wrote the manuscript. All authors contributed to the article and approved the submitted version.

## Conflict of Interest

The authors declare that the research was conducted in the absence of any commercial or financial relationships that could be construed as a potential conflict of interest.
