# Supplementary material for: Maimendong Decoction Improves Pulmonary Function in Rats With Idiopathic Pulmonary Fibrosis by Inhibiting Endoplasmic Reticulum Stress in AECIIs
Source: Front Pharmacol. 2020 Aug 14;11:1262. doi: 10.3389/fphar.2020.01262 (PMC7466437; doi:10.3389/fphar.2020.01262)
Supplement: Supplementary file 1 [file DataSheet_1.docx]

**Supplementary Table 1|** Detailed information of the crude drugs composed in MMDD.

| **No.** | **Drug Name** | **Authentication** | **Voucher specimen** |
| --- | --- | --- | --- |
| MMDD-A | Ophiopogonis Radix | Roots of Ophiopogon japonicus (Thunb.) Ker Gawl. | 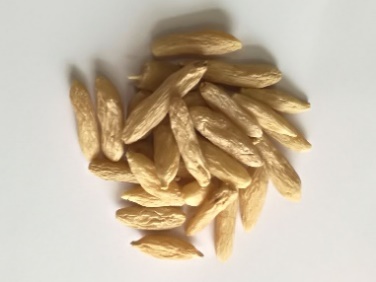 |
| MMDD-B | Pinelliae Rizoma Praeparatum Cum Alumine | Roots of Pinellia ternata (Thunb.) Breit. | 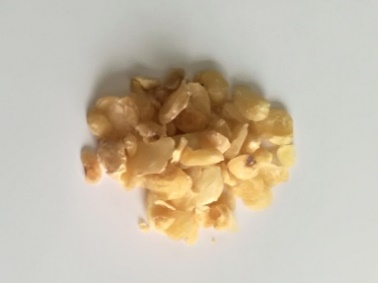 |
| MMDD-C | Glycyrrhizae Radix Et Rhizoma Praeparata Cum Melle | Stems of Glycyrrhiza uralensis Fisch. ex DC. | 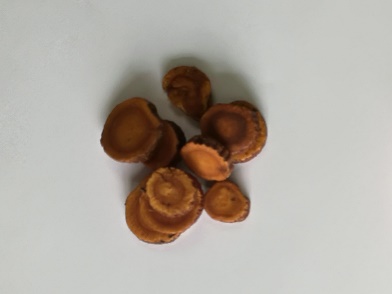 |
| MMDD-D | Ginseng Radix Et Rhizoma | Rhizomes of Panax ginseng C. A. Mey. | 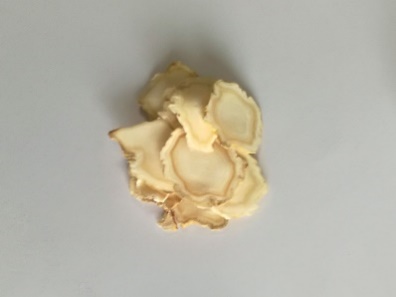 |
| MMDD-E | Oryza sativa subsp. japonica S.Kato | Seeds of Oryza sativa L. | 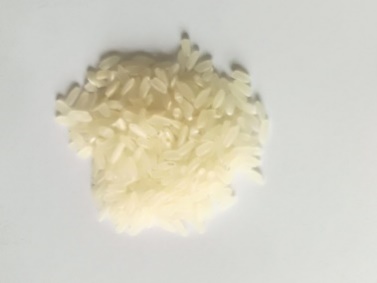 |
| MMDD-F | Jujubae Fructus | Fruits of Ziziphus jujuba Mill. | 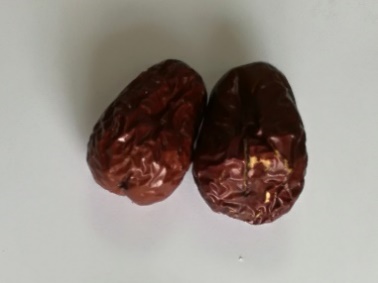 |

**Supplementary Table 2|** The procedure of elution

| Time(min) | A(v%) | B(v%) |
| --- | --- | --- |
| 0 | 100 | 0 |
| 1.8 | 100 | 0 |
| 7 | 40 | 60 |
| 9 | 0 | 100 |
| 11 | 0 | 100 |
| 11.1 | 100 | 0 |
| 13 | 100 | 0 |
